# Comparative Genomic-Based Study of Reproduction-Related Genes in Three Fruit Fly Species

**DOI:** 10.3389/fgene.2022.893695

**Published:** 2022-05-18

**Authors:** Yinggu Wu, Yuyang Lian, Sihua Peng, Aqiang Wang, Heming Yang, Jinlei Li, Shuyan Yang, Shihao Zhou

**Affiliations:** ^1^ College of Plant Protection, Hainan University, Haikou, China; ^2^ Sanya Nanfan Research Institute of Hainan University, Sanya, China

**Keywords:** comparative genomics, fruit fly, reproduction, vitellogenin, fatty acid metabolism

## Abstract

*Zeugodacus cucurbitae* (Coquillett), *Bactrocera dorsalis* (Hendel), and *Ceratitis capitata* (Wiedemann) are important pests of fruit and vegetable crops and are difficult to control because of their rapid reproduction rate and egg production. To investigate the key genes regulating reproduction in three fruit fly species, we selected genomic information of three fruit fly species, screened specific genes and single-copy homolog genes, and performed KEGG and GO enrichment analysis on specific genes and single-copy homolog genes of the strong positive select (SP); the results showed that *Z. cucurbitae* (Coquillett), *B. dorsalis* (Hendel), and *C. capitata* (Wiedemann) had seven, 11, and one *Vitellogenin*-related genes, respectively; *Z. cucurbitae* (Coquillett) had 84 specific genes enriched in immune system-related pathways; *B. dorsalis* (Hendel) had 1,121 specific genes enriched in signaling pathways related to cell growth and differentiation; *C. capitata* (Wiedemann) had 42 specific genes enriched in the degradation and metabolism pathways of exogenous organisms; *Z. cucurbitae* (Coquillett) may have a stronger immune system; *B. dorsalis* (Hendel) has a faster developmental and reproductive rate; and *C. capitata* (Wiedemann) has a higher detoxification capacity. Only one SP single-copy homolog gene (gene name: very long-chain specific acyl-CoA dehydrogenase, mitochondrial) is enriched in the fatty acid metabolic pathway in both *Z. cucurbitae* (Coquillett) and *B. dorsalis* (Hendel) as well as in *Z. cucurbitae* (Coquillett) and *C. capitata* (Wiedemann). This study provides a molecular basis for studying the reproductive mechanisms of three fruit fly species and provides a scientific basis for developing effective control strategies for fruit flies.

## Introduction

Fruit flies (Diptera: Tephritidae) have a large number of species, with about 500 genera and 4,500 species known worldwide, of which about 1,500 species may be associated with various fruits and more than 250 species are of economic importance (Ao et al., 2019). *Zeugodacus cucurbitae* (Coquillett), *Bactrocera dorsalis* (Hendel), and *Ceratitis capitata* (Wiedemann) are important quarantine pest fruit flies that are widely distributed in the tropical, subtropical, and temperate regions, and *Z. cucurbitae* (Coquillett) originated in India and mainly affects plants of the Cucurbitaceae and can parasitize more than 125 species of plants (He et al., 2019); *B. dorsalis* (Hendel) originated in Southeast Asia and affects more than 450 species of fruits and vegetables (Su et al., 2021); *C. capitata* (Wiedemann) originated in the western region of sub-Saharan Africa and affects more than 260 species of fruits and vegetables, including Solanaceae (Papanicolaou et al., 2016). There are three fruit flies species that are highly reproductive and can occur in multiple generations a year. The female fruit flies pierce the ovipositor into the surface of melon and fruit to lay eggs, and the larvae hatch and feed inside the melon and fruit, leaving puncture holes on the surface of the affected fruit and deformation around the puncture wounds, and in severe cases, the fruit rots and causes fruit drop, resulting in huge economic losses (Wu et al., 2021). A comparative analysis of the genomes of the three fruit flies species can explore the physiological characteristics of development and reproduction of the three species and provide a scientific basis for the development of effective fruit flies control strategies.

Comparative genomics is a discipline that compares known genes and genomic structures based on genome mapping and sequencing to understand the gene function, expression mechanisms, and species evolution. (Dai and Li, 2004). Comparative genomes may be studied for the same species with different genetic backgrounds or for different species at similar developmental stages. The comparative genomic analysis of *Lactobacillus plantarum* from different sources revealed that *L. plantarum* of animal intestinal origin had better amino acid transport capacity and *L. plantarum* strains attached to plant surfaces had better carbohydrate utilization capacity (Liu et al., 2021). Zhao et al. (2013) comparatively analyzed the genomes of common oil tea and tea and found 131 homologous gene pairs with Ka/Ks values greater than 1, indicating that these homologous genes have been subjected to positive selection during the evolution of the subgenus Camellia and tea and have evolved rapidly in the recent past. Libbrecht and Keller (2015) comparatively analyzed the genomes of *Bombus terrestris* and *Bombus impatiens* and found high similarities; then, the genome of bumblebees and honeybees were compared, concluding that the genome of bumblebees is characterized by a lower level of sociality than that of honeybees.

Vitellogenin (Vg) is a large-molecule lipoprotein of the phospho-glycine class. After synthesis in the fat body, Vg is secreted into the hemolymph, where it is normally taken up by the developing oocyte as the main nutrient in the egg, but in the absence of food, increased feeding pressure, failure to find an egg-laying site, or the lack of males to mate with, egg absorption occurs, and Vg in the egg is degraded and released into the hemolymph as a nutrient to maintain individual development. The primary function of Vg is to be a source of energy for egg production and development of insects, including amino acids, carbohydrates, fats, phosphates, vitamins, and sulfates (Sappington and Raikhel, 1998). In recent years, it has been shown that insect Vg is a multi-potent protein that not only provides nutrition for embryonic development but also has functions such as stress and antioxidant resistance (Corona et al., 2007), climate adaptation (Amdam et al., 2005), ovary activation, lifespan regulation, and wing differentiation (Page-Jr and Amdam, 2007). In particular, in social insects, it regulates the social division of labor and hierarchy (Nelson et al., 2007). In this study, the genomes of *Z. cucurbitae* (Coquillett), *B. dorsalis* (Hendel), and *C. capitata* (Wiedemann) were comparatively analyzed to further investigate the mechanisms of regulating reproduction in the Vg-related genes of the three species and to provide new ideas for the control of the three fruit fly species.

## Materials and Methods

### Acquisition of Basic Genomic Information

Basic genome information for *Z. cucurbitae* (Coquillett), *B. dorsalis* (Hendel), and *C. capitata* (Wiedemann) was obtained from the National Center for Biotechnology Information (NCBI) (https://www.ncbi.nlm.nih.gov/).

### Genome Analysis

The genomic data of *Z. cucurbitae* (Coquillett), *B. dorsalis* (Hendel), and *C. capitata* (Wiedemann) were used to identify homologous genes using blastp in combination with OrthoMCL. After obtaining homologous and specific gene families, the number of genes contained in each gene family (the number of gene family copies) was counted in each of the three fruit fly species, and the gene families specific to the three fruit fly species were identified and subjected to functional annotation, KEGG enrichment analysis, and GO enrichment analysis. The gene families corresponding to homologous genes in the three fruit fly species were classified into two gene sets, single-copy homologous genes, and multi-copy homologous genes, and the Ka/Ks values of single-copy homologous genes were calculated, and the single-copy homologous genes were classified into the strong positive select (SP), weak positive select (WP), and negative select (NG) genes according to the Ka/Ks values, and KEGG enrichment analysis and GO enrichment analysis were performed on the SP single-copy homolog genes.

### KEGG Enrichment Analysis

Within an organism, different genes coordinate their biology with each other, and pathway-based analysis helps to further understand the biological functions of genes. KEGG is the main public database on pathways. The pathway significant enrichment analysis uses the KEGG pathway as the unit and applies a hypergeometric test to identify pathways that are significantly enriched in species-specific genes when compared to the entire genome. The *p*-value of this hypothesis test is calculated as follows:

$$\text{p}=1-\sum_{i=0}^{\text{m}-1}\frac{\left(\begin{array}{c} M\\ i \end{array}\right)\left(\begin{array}{c} N-M\\ n-\text{i} \end{array}\right)}{\left(\begin{array}{c} N\\ n \end{array}\right)},$$

where N is the number of genes with pathway annotation in all unigenes, n is the number of specifically expressed genes in N, M is the number of genes annotated with a specific pathway in all unigenes, and m is the number of specific genes expressed with a specific pathway annotation. After correction for multiple testing, pathways with Q-value ≤ 0.05 were selected as those significantly enriched in specific genes. The q-value and FDR are similar, both being a correction for the *p*-value. The Q-value of a particular hypothesis test is the minimum of the FDR, and the hypothesis test can be considered significant under these FDR. Enrichment by pathway significance identifies the most important biochemical metabolic pathways and signal transduction pathways involved in specific genes.

### GO Enrichment Analysis

Gene Ontology (GO) is an international standardized gene function classification system that provides a dynamically updated set of controlled vocabulary to comprehensively describe the properties of genes and gene products in an organism. GO has a total of three ontologies, which describe the molecular function of a gene, the cellular component, and the biological process involved. Each term corresponds to an attribute.

GO function significant enrichment analysis provides GO function entries that are significantly enriched in specific genes after comparison with reference genes and screens which biological functions are significantly associated with specific genes. This analysis first maps all specific genes to each term of the Gene Ontology database (http://www.geneontology.org/) and counts the number of genes per term to obtain a list of genes with a particular GO function and a count of the number of genes. A hypergeometric test was then applied to identify GO entries that were significantly enriched in differentially expressed genes compared to the whole genomic background, and the *p*-value of this hypothesis test was calculated as follows:

$$\text{p}=1-\sum_{i=0}^{\text{m}-1}\frac{\left(\begin{array}{c} M\\ i \end{array}\right)\left(\begin{array}{c} N-M\\ n-\text{i} \end{array}\right)}{\left(\begin{array}{c} N\\ n \end{array}\right)},$$

where N is the number of genes with GO annotation in all genes, n is the number of species-specific genes in N, M is the number of genes annotated with a particular GO term in all genes, and m is the number of species-specific genes annotated with a particular GO term. The calculated *p*-value was corrected by Bonferroni with a threshold value of corrected *p*-value ≤ 0.05, and the GO term meeting this condition was defined as a GO term significantly enriched in species-specific genes.

### Ka/Ks of Single-Copy Homologous Genes

In genetics, Ka/Ks or dN/dS indicates the ratio between the rate of non-synonymous substitutions (Ka) and the rate of synonymous substitutions (Ks). This ratio can determine whether there is a selective pressure acting on this protein-coding gene. It is generally accepted that synonymous mutations are not subjected to natural selection, whereas non-synonymous mutations are subjected to the action of natural selection. In evolutionary analysis, it is of interest to know the rate at which synonymous and non-synonymous mutations occur. The following parameters are commonly used: the frequency of synonymous mutations (Ks), the frequency of non-synonymous mutations (Ka), and the ratio of the rate of non-synonymous mutations to the rate of synonymous mutations (Ka/Ks). Single-copy homologous gene protein sequences from different species or subspecies were subjected to multiple sequence alignment by using muscle (http://www.drive5.com/muscle), and then the Ka ratio between each single-copy homologous gene was calculated based on the nucleic acid sequence corresponding to the protein sequence. The Ka, Ks, and Ka/Ks values between homologous genes were calculated using the KaKs_Calculator Toolbox (version 2.0).

## Results

### Basic Information of the Genome

The basic information of the genomic data of the three fruit fly species is shown in Table 1.

**TABLE 1:** Transcriptome data of *Zeugodacus cucurbitae* (Coquillett), *Bactrocera dorsalis* (Hendel), and *Ceratitis capitata* (Wiedemann).

Species	Transcriptome	N50	GC%	Max len	Min len	CDS	Protein
*Zeugodacus cucurbitae* (Coquillett)	24202	4424	41.8892	42496	161	24202	24202
*Bactrocera dorsalis* (Hendel)	20820	3845	41.3035	57701	89	20820	20820
*Ceratitis capitata* (Wiedemann)	22936	4147	41.4697	64084	210	22936	22936

**FIGURE 1:**
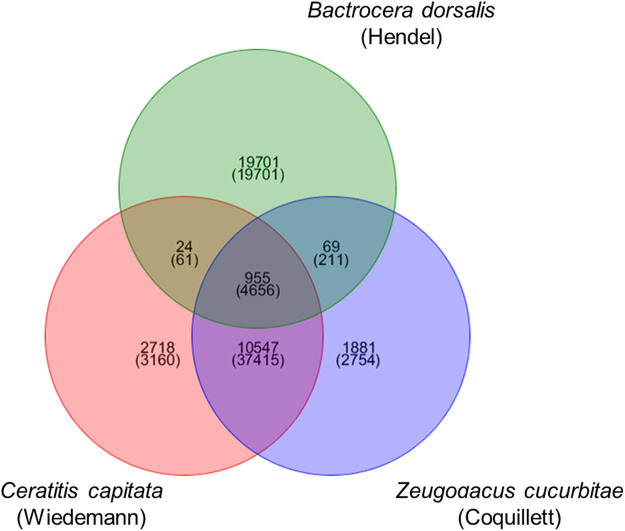
Venn diagram of the gene family of *Zeugodacus cucurbitae* (Coquillett), *Bactrocera dorsalis* (Hendel), and *Ceratitis capitata* (Wiedemann). Note: the upper number in each section represents the number of gene families, and the lower number represents the number of genes.

For the genomes of the three fruit fly species, blastp was used in conjunction with OrthoMCL to identify homologous genes among the three species. The sequences were matched two by two, and pairs with an E-value less than 1e-5 and a coverage of more than 30% were considered homologous genes in the Blast comparison. OrthoMCL was used to assign homologous genes to the same family. As shown in Figure 1, there were 4,656 homologous genes and 955 gene families in the three fruit fly species, 211 and 69 homologous genes and gene families in *Z cucurbitae* (Coquillett) and *B. dorsalis* (Hendel), 37,415 and 10,547 homologous genes and gene families in *Z cucurbitae* (Coquillett) and *C. capitata* (Wiedemann), and 61 and 24 homologous genes and gene families in *B. dorsalis* (Hendel) and *C. capitata* (Wiedemann). *Z cucurbitae* (Coquillett) had 2,754 specific genes and 1,881 gene families, *B. dorsalis* (Hendel) had 19701 specific genes and 19701 gene families, and *C. capitata* (Wiedemann) had 3,160 specific genes and 2,718 gene families.

## Analysis of Specific Genes

### KEGG Enrichment Analysis of Specific Genes

**FIGURE 2:**
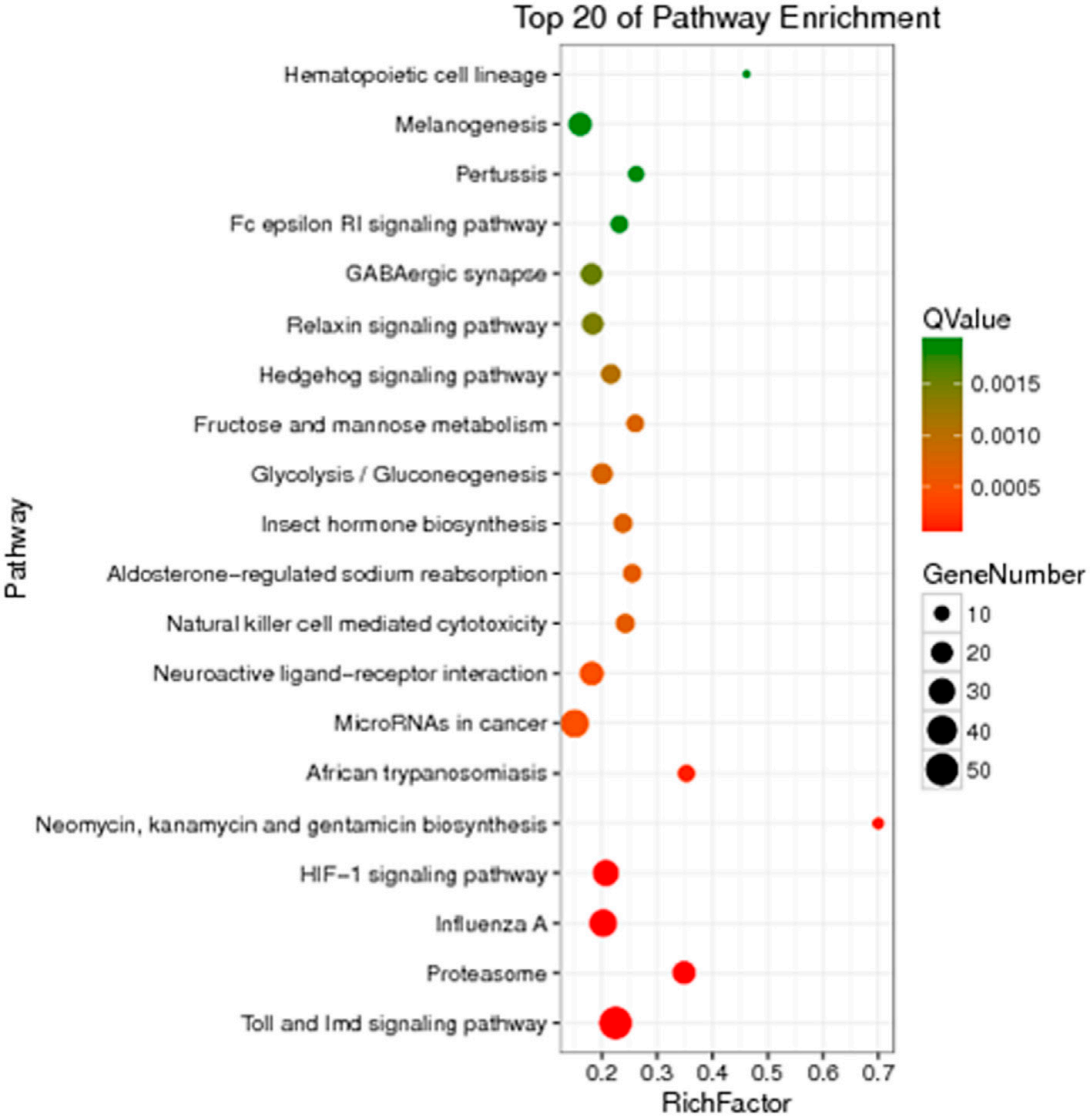
KEGG enrichment analysis results of specific genes of *Zeugodacus cucurbitae* (Coquillett).

**FIGURE 3:**
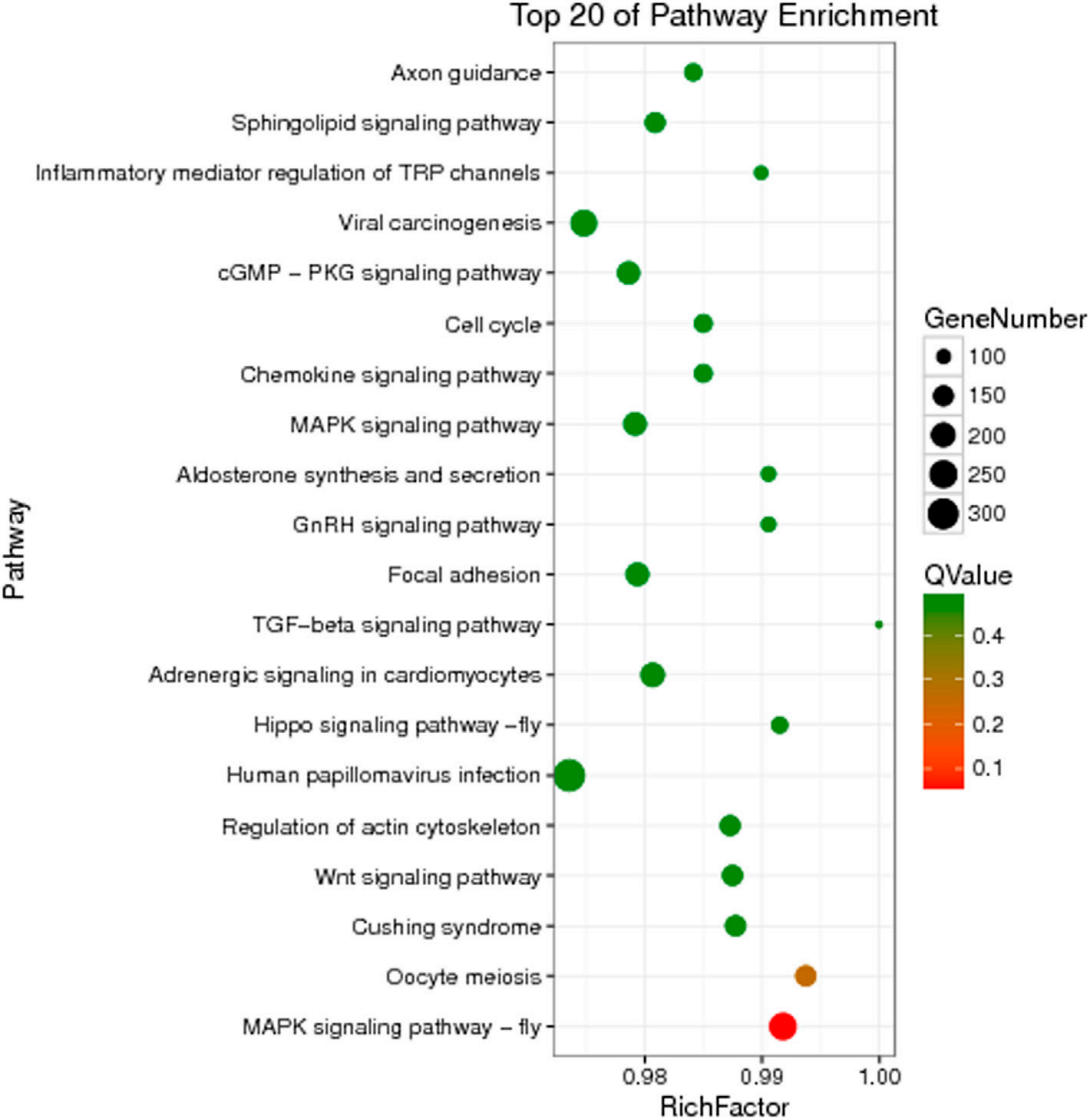
KEGG enrichment analysis results of specific genes of *Bactrocera dorsalis* (Hendel).

**FIGURE 4:**
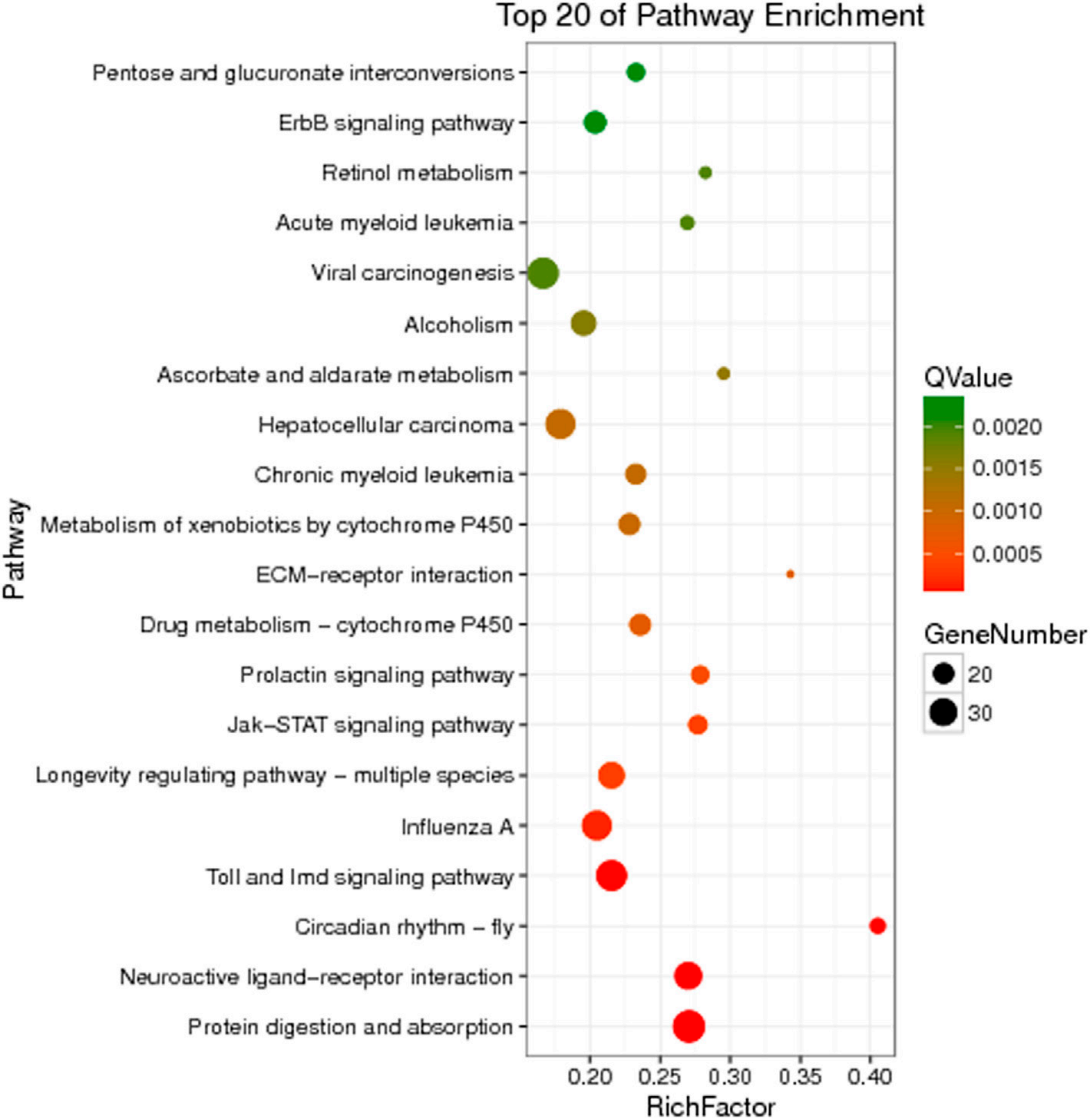
KEGG enrichment analysis results of specific genes of *Ceratitis capitata* (Wiedemann).

The unigenes of *Z cucurbitae* (Coquillett), *B. dorsalis* (Hendel), and *C. capitata* (Wiedemann) were annotated to the KEGG database for comparison, and their specific gene KEGG enrichment analysis bubble maps were obtained to identify the pathways that were significantly enriched in specific genes. The highest gene abundance in *Z. cucurbitae* (Coquillett) was found for the Toll and Imd signaling pathway with about 50 genes, followed by MicroRNAs in cancer and influenza A, all three with Q values less than 0.0005. Among all metabolic pathways in *Z. cucurbitae* (Coquillett), the largest enrichment factor was neomycin, kanamycin, and gentamicin biosynthesis with an enrichment factor of about 0.7, followed by hematopoietic cell lineage, with an enrichment factor of about 0.46 (Figure 2). The highest gene abundance was found in *B. dorsalis* (Hendel) with human papillomavirus infection, enriched with 331 genes, followed by the MAPK signaling pathway—fly MAPK and viral carcinogenesis. Among all metabolic pathways in *B. dorsalis* (Hendel), the largest enrichment factor is the TGF-beta signaling pathway with an enrichment factor of about 1, followed by oocyte meiosis and the Hippo signaling pathway—fly (Figure 3). The highest KEGG enrichment results for *C. capitata* (Wiedemann) were found for protein digestion and absorption (39 genes), followed by viral carcinogenesis and the Toll and Imd signaling pathway. Among all metabolic pathways in *C. capitata* (Wiedemann), the largest enrichment factor is circadian rhythm—fly with an enrichment factor of about 0.42, followed by ECM-receptor interaction with an enrichment factor of about 0.34 (Figure 4).

### GO Enrichment Analysis of Specific Genes

**FIGURE 5:**
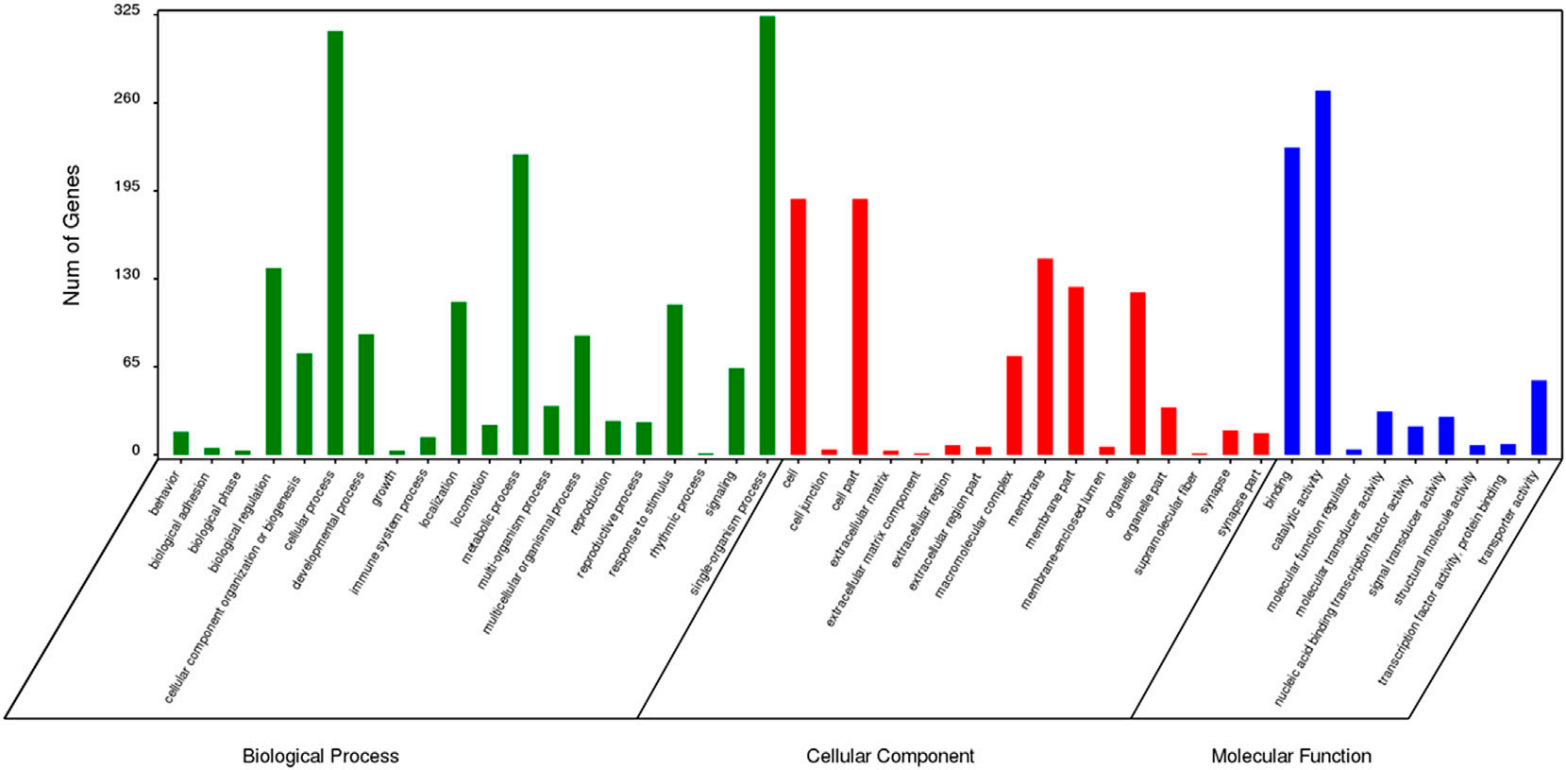
GO function classification of specific genes of *Zeugodacus cucurbitae* (Coquillett).

**FIGURE 6:**
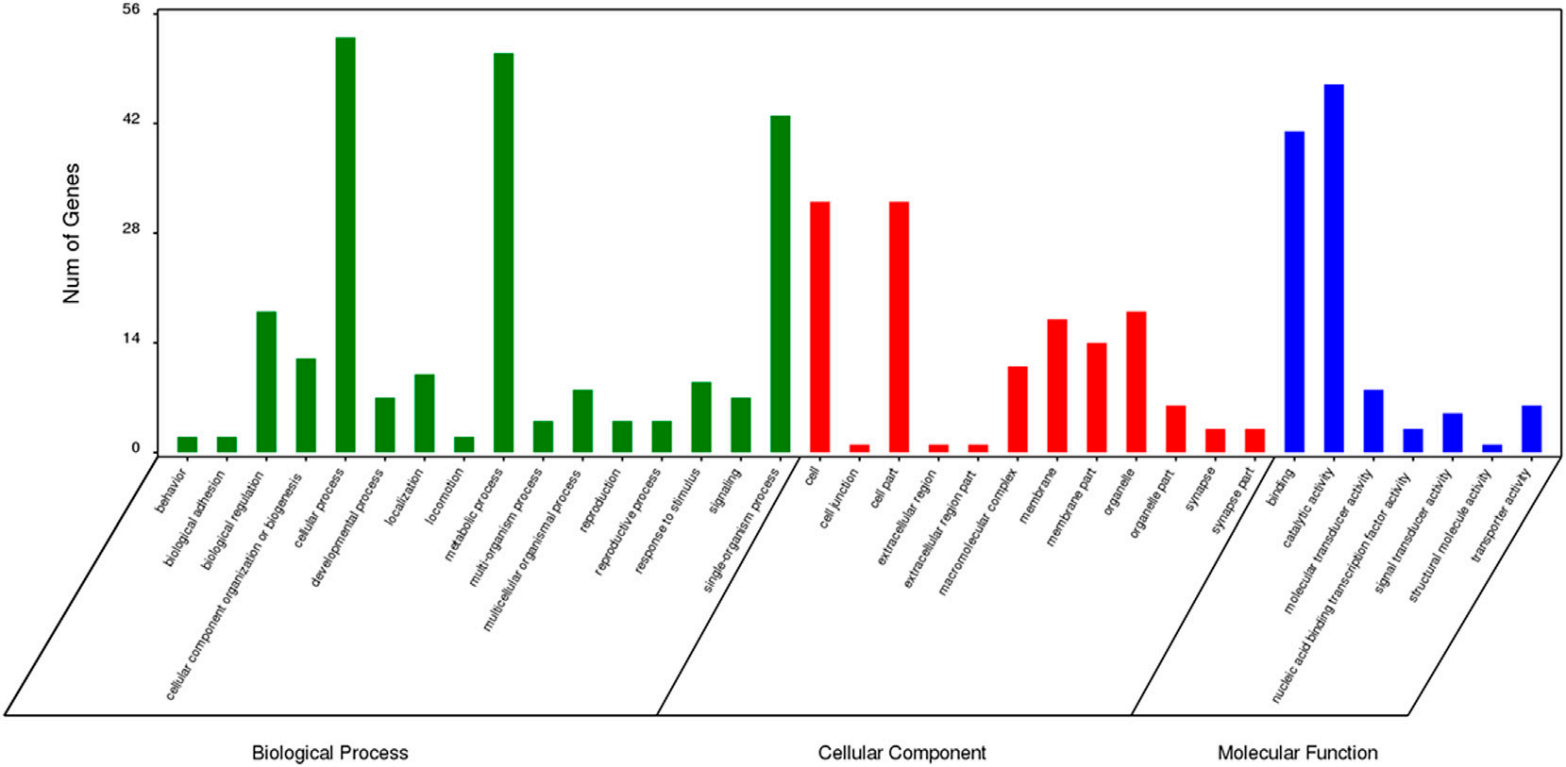
GO function classification of specific genes of *Bactrocera dorsalis* (Hendel).

**FIGURE 7:**
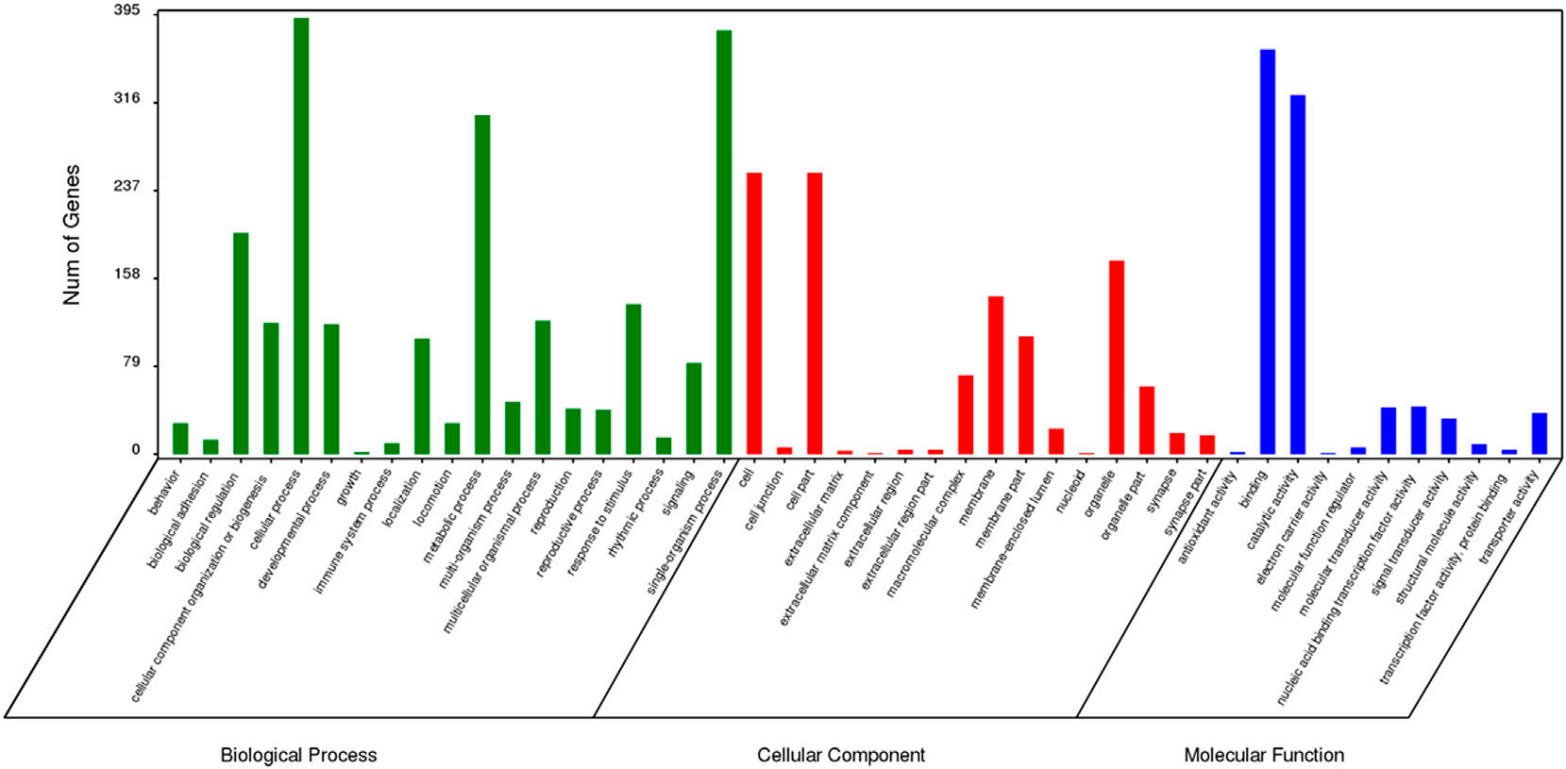
GO function classification of specific genes of *Ceratitis capitata* (Wiedemann).

The results of GO enrichment analysis for genes specific to *Z. cucurbitae* (Coquillett), *B. dorsalis* (Hendel), and *C. capitata* (Wiedemann) are shown in Figures 5–7. In the broad category of biological processes, all three species were enriched for more genes in metabolic processes, intracellular processes, and single-tissue processes, and *Z. cucurbitae* (Coquillett) was enriched most in the broad category of cellular components in the pathways of membranes, cells, and cellular components. *B. dorsalis* (Hendel) and *C. capitata* (Wiedemann) were most enriched in the cellular component major category for cellular, cellular component, and organelle pathways, and in the molecular function major category, all three species were more catalytically active and binding enriched for genes.

## Analysis of Single-Copy Genes

### Calculation of Ka/Ks for Single-Copy Homologous Genes

**FIGURE 8:**
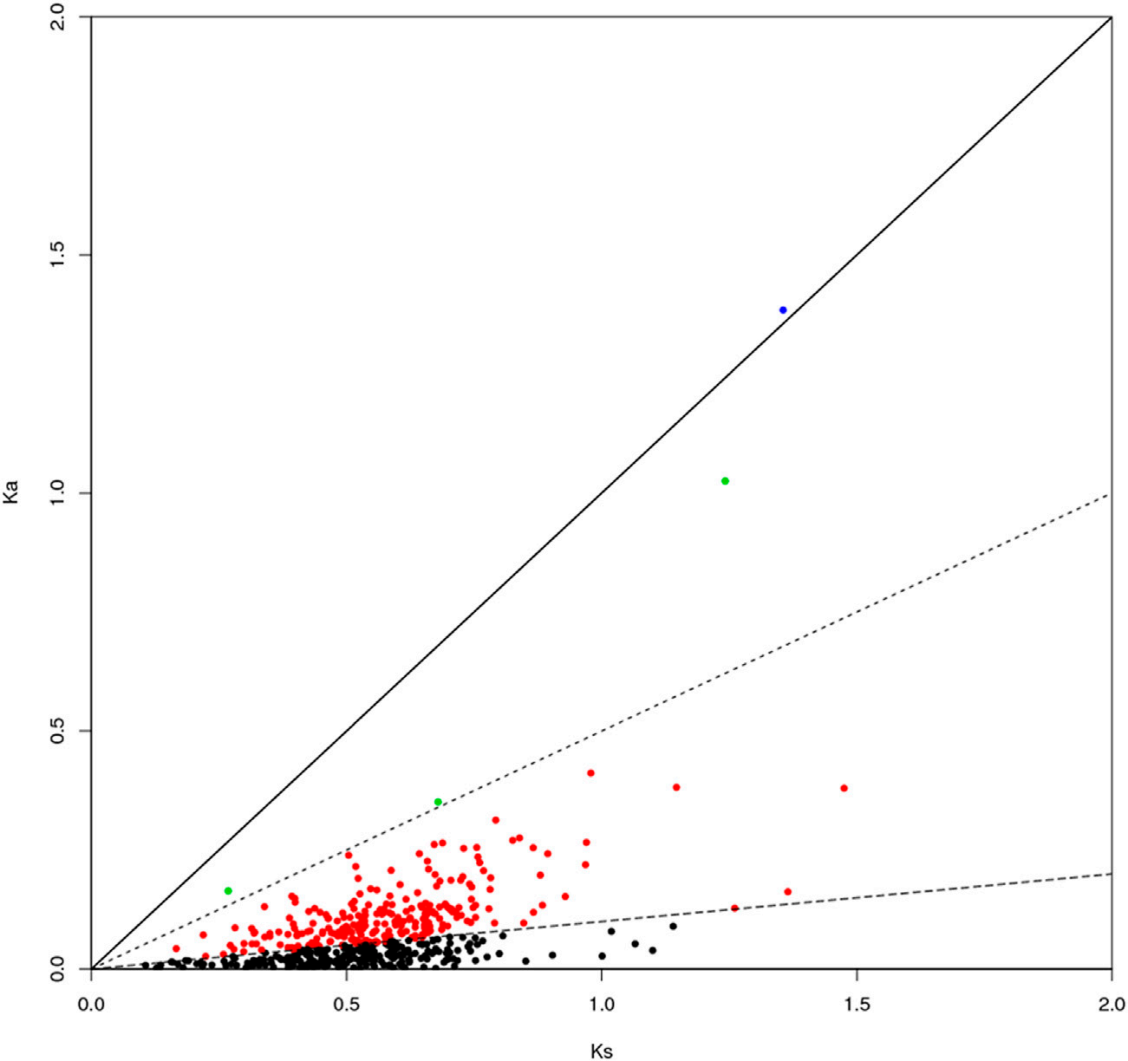
Scatter plot of Ka/Ks obtained by comparing *Zeugodacus cucurbitae* (Coquillett) and *Bactrocera dorsalis* (Hendel). Note: the region of blue spot: strong positive select genes; the region of green spot: weak positive selection genes; the region of black spot: purification selection genes.

**FIGURE 9:**
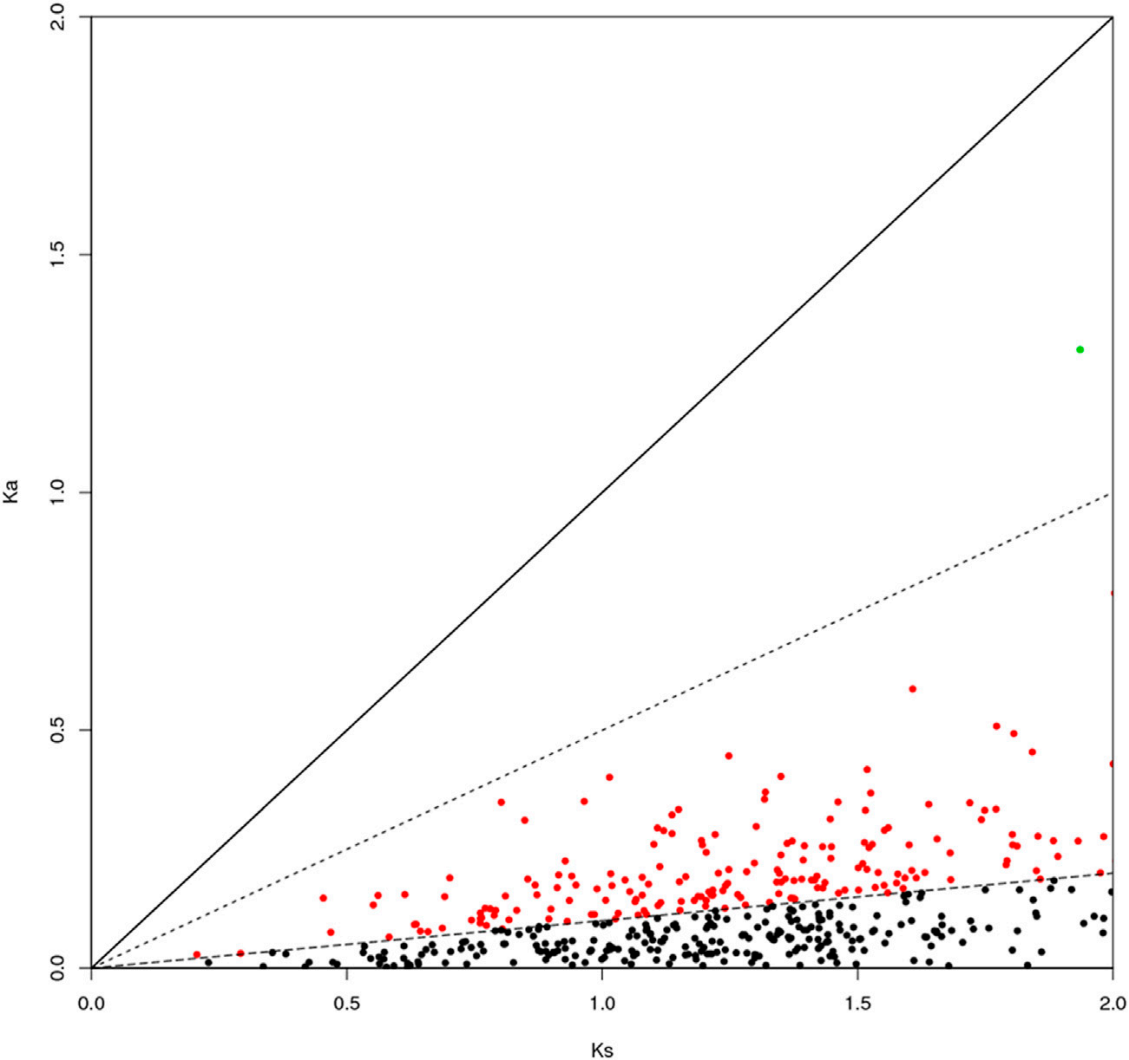
Scatter plot of Ka/Ks obtained by comparing *Zeugodacus cucurbitae* (Coquillett) and *Ceratitis capitata* (Wiedemann). Note: the region of blue spot: strong positive select genes; the region of green spot: weak positive selection genes; the region of black spot: purification selection genes.

**FIGURE 10:**
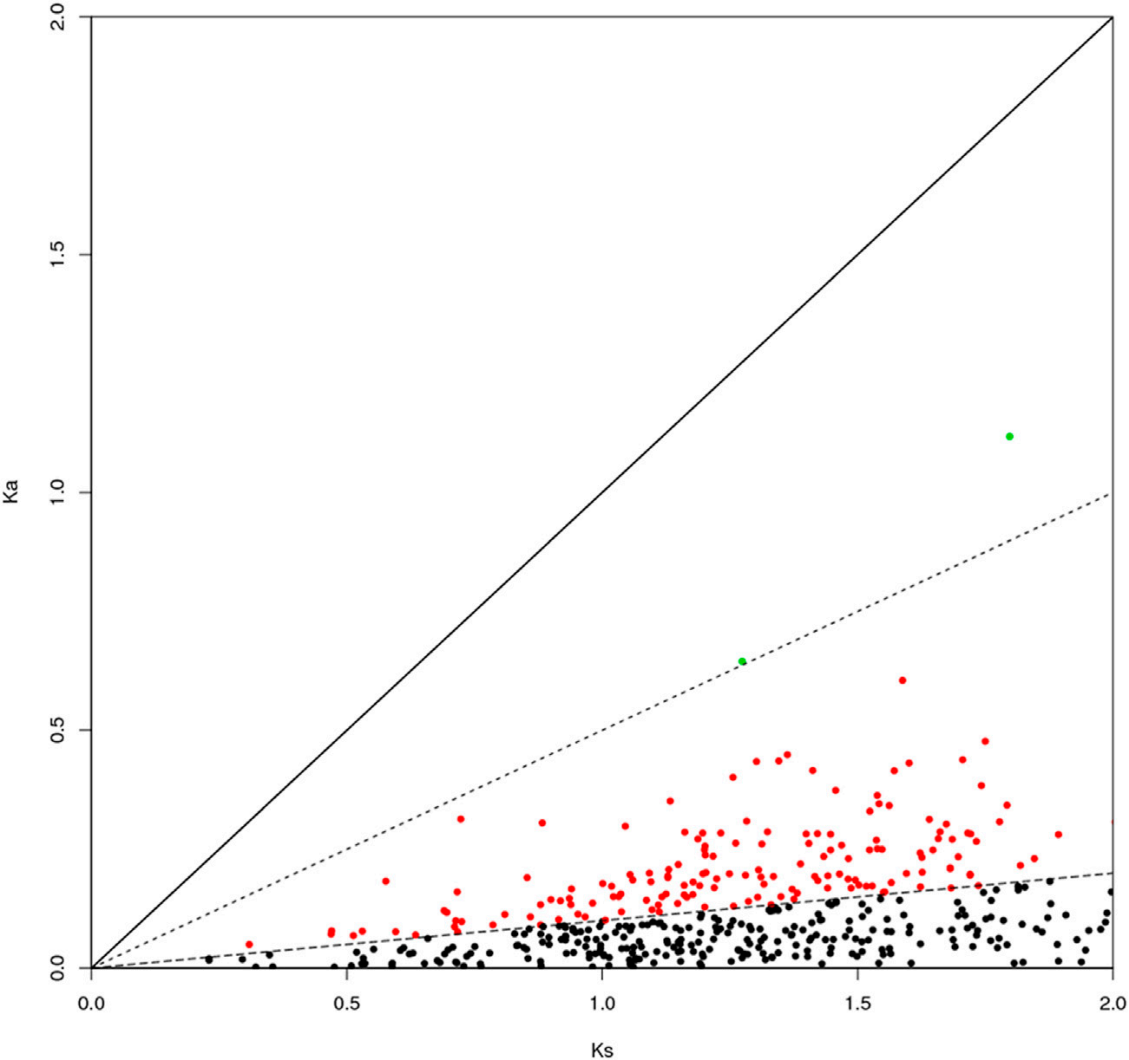
Scatter plot of Ka/Ks obtained by comparing *Bactrocera dorsalis* (Hendel) and *Ceratitis capitata* (Wiedemann). Note: the region of blue spot: strong positive select genes; the region of green spot: weak positive selection genes; the region of black spot: purification selection genes.

The genes can be classified according to the Ka/Ks values of single-copy homologous genes. *Z. cucurbitae* (Coquillett) and *B. dorsalis* (Hendel) have 508 homologous unigene pairs (Figure 8); and two unigenes have Ka/Ks ratios greater than 1 (Table 2), and the remaining 506 have Ka/Ks ratios less than or equal to 1. *Z. cucurbitae* (Coquillett) and *C. capitata* (Wiedemann) have 519 homologous unigene pairs (Figure 9); and two unigenes have Ka/Ks ratios greater than 1 (Table 3). *B. dorsalis* (Hendel) and *C. capitata* (Wiedemann) have 517 homologous unigene pairs with Ka/Ks ratios less than 1 (Figure 10). Because most non-synonymous mutations are deleterious and single-copy homologous genes have a low tolerance for deleterious mutations on them due to the lack of copies, most genes have small Ka/Ks values. To identify positively selected genes that are neutralized (masked) by a large number of deleterious mutation sites, genes with Ka/Ks > 1 are defined as SP, genes with Ka/Ks before 0.5 and 1 are WP, and genes with Ka/Ks < 0.1 are NG (purifying selection).

**TABLE 2:** Strong positive select genes of *Zeugodacus cucurbitae* (Coquillett) and *Bactrocera dorsalis* (Hendel).

Sequence	Gene name	Ka	Ks	Ka/Ks
XM_011197361.2	Cuticle protein19	1.3841	1.35519	1.02134
XM_011196673.2	Very long-chain specific acyl-CoA dehydrogenase, mitochondrial	2.49745	1.75193	1.42554

**TABLE 3:** Strong positive select genes of *Zeugodacus cucurbitae* (Coquillett) and *Ceratitis capitata* (Wiedemann).

Sequence	Gene name	Ka	Ks	Ka/Ks
XM_011196673.2	Very long-chain specific acyl-CoA dehydrogenase, mitochondrial	3.24129	2.35129	1.37852
XM_029040384.1	Queuosine salvage protein	2.73845	2.27928	1.20145

### Functional Enrichment Analysis of Selection-Related Genes

The KEGG enrichment analysis showed that only one gene was enriched among the single-copy homolog genes of *Z. cucurbitae* (Coquillett) and *B. dorsalis* (Hendel), and the enriched pathways were fatty acid degradation, fatty acid metabolism, and lipid metabolism (Table 4), and the KEGG enrichment results of the single-copy homolog genes of *Z. cucurbitae* (Coquillett) and *C. capitata* (Wiedemann) were similar to those of *Z. cucurbitae* (Coquillett) and *B. dorsalis* (Hendel) (Table 5). GO enrichment analysis showed that genes were enriched in the three broad categories of biological processes, molecular functions, and cellular components, and in the broad category of biological processes of *Z. cucurbitae* (Coquillett) and *B. dorsalis* (Hendel), genes were enriched in the GO:0044699-single-organism process. Among the molecular functional categories, genes are enriched in GO: 0005488-binding, GO:0003824-catalytic activity, and GO:0005198-structural molecule activity, while the cellular components are enriched in GO:0005623-cell, GO:0044464-cell part, and GO:0043226-organelle (Table 6). In the biological process category of *Z. cucurbitae* (Coquillett) and *C. capitata* (Wiedemann), genes are enriched in the GO:0008152-metabolic process and GO:0044699-single-organism process; in the molecular function category, genes are enriched in GO:0005488-binding and GO:0003824-catalytic activity; and the cellular component is enriched in GO:0005623-cell, GO:0044464-cell part, and GO:0032991-macromolecular complex (Table 7). In the biological process category of *B. dorsalis* (Hendel) and *C. capitata* (Wiedemann), genes are enriched in the GO:0008152-metabolic process and GO:0044699-single-organism process; in the molecular function category, genes are enriched in GO:0005488-binding and GO:0003824-catalytic activity; and in the cellular component, genes are enriched in GO:0005623-cell and GO:0044464-cell part (Table 8).

**TABLE 4:** KEGG enrichment for strong positive select genes by comparing *Zeugodacus cucurbitae* (Coquillett) and *Bactrocera dorsalis* (Hendel).

Pathway	Gene number (ratio)	*p*-value	Q-value	Pathway ID
Fatty acid degradation	1 (100%)	0.008233	0.016034	ko00071
Fatty acid metabolism	1 (100%)	0.010689	0.016034	ko01212
Metabolic pathways	1 (100%)	0.227936	0.227936	ko01100

**TABLE 5:** KEGG enrichment for strong positive select genes by comparing *Zeugodacus cucurbitae* (Coquillett) and *Ceratitis capitata* (Wiedemann).

Pathway	Gene number (ratio)	*p*-value	Q-value	Pathway ID
Fatty acid degradation	1 (100%)	0.011278	0.021368	ko00071
Fatty acid metabolism	1 (100%)	0.014245	0.021368	ko01212
Metabolic pathways	1 (100%)	0.236385	0.236385	ko01100

**TABLE 6:** GO enrichment for strong positive select genes by comparing *Zeugodacus cucurbitae* (Coquillett) and *Bactrocera dorsalis* (Hendel).

GO classification	GO ID	Function description	Gene number (Ratio)	*p*-value
Biological process	GO:0044699	Single-organism process	2 (100%)	0.366855
	GO:0005488	Binding	1 (50%)	0.804991
Molecular function	GO:0003824	Catalytic activity	1 (50%)	0.812277
	GO:0005198	Structural molecule activity	1 (50%)	0.043279
	GO:0005623	Cell	16 (64%)	0.520990
Cellular component	GO:0044464	Cell part	16 (64%)	0.520990
	GO:0043226	Organelle	11 (44%)	0.533010

**TABLE 7:** GO enrichment for strong positive select genes by comparing *Zeugodacus cucurbitae* (Coquillett) and *Ceratitis capitata* (Wiedemann).

GO classification	GO ID	Function description	Gene number (Ratio)	*p*-value
Biological process	GO:0008152	Metabolic process	1 (100%)	0.514965
	GO:0044699	Single-organism process	1 (100%)	0.605713
Molecular function	GO:0005488	Binding	1 (100%)	0.558363
	GO:0003824	Catalytic activity	1 (100%)	0.566690
Cellular component	GO:0005623	Cell	4 (66.7%)	0.592865
	GO:0044464	Cell part	4 (66.7%)	0.592865
	GO:0032991	Macromolecular complex	4 (66.7%)	0.041778

**TABLE 8:** GO enrichment for strong positive select genes by comparing *Bactrocera dorsalis* (Hendel) and *Ceratitis capitata* (Wiedemann).

GO classification	GO ID	Function description	Gene number (Ratio)	*p*-value
Biological process	GO:0008152	Metabolic process	11 (44%)	0.971057
	GO:0044699	Single-organism process	9 (36%)	0.994638
Molecular function	GO:0005488	Binding	5 (25%)	0.997408
	GO:0003824	Catalytic activity	9 (45%)	0.962511
Cellular component	GO:0005623	Cell	3 (30%)	0.999294
	GO:0044464	Cell part	3 (30%)	0.999294

## *Vitellogenin* - Related Gene Specificity Analysis

Vg production and uptake play an important role in the egg-laying behavior of fruit flies. Among the genes specific to *Z. cucurbitae* (Coquillett), 7 are associated with *Vg* genes, 5 are associated with *Vg-1* genes and 2 with *Vg-3* genes. *B. dorsalis* (Hendel) had 11 *Vg*-related specific genes, with four associated with *Vg-1* genes, one associated with *Vg-2* genes, one associated with *Vg-3* genes, and five associated with other *Vg* genes. *C. capitata* (Wiedemann) had only 1 *Vg-1* gene-related specific gene (Table 9). Vg is the precursor of vitellin (Vn), which is the main egg storage protein and provides amino acids, fat, carbohydrates, phosphorus, sulfur, and trace elements for later embryonic development after accumulation in the egg (Huo et al., 2018), and among the genes specific to the three fruit fly species, *B. dorsalis* (Hendel) had significantly more number and variety of *Vg*-related genes than *Z. cucurbitae* (Coquillett) and *C. capitata* (Wiedemann), indicating that *B. dorsalis* (Hendel) can provide more energy for the reproduction of offspring, giving it characteristics such as faster reproduction and larger egg production. Among the genes specific to *B. dorsalis* (Hendel), there are 3 *Vg-A2*, 1 *Vg-like*, and 1 *Vg-6* genes, and studies have shown that Vg-A2 and Vg-like are precursors of Vg (Keller et al., 1995; Richards, 2019), and Vg-6 is a Vg synthesized and secreted in the intestine during the egg-laying stage (Nakamura et al., 1999), and the functions of the three *Vg-*related genes need to be further investigated.

**TABLE 9:** Number of *Vitellogenin*-*related* genes in specific genes of *Zeugodacus cucurbitae* (Coquillett), *Bactrocera dorsalis* (Hendel), and *Ceratitis capitata* (Wiedemann).

Species	*Vitellogenin-1*	*Vitellogenin-2*	*Vitellogenin-3*	Others	Total
*Zeugodacus cucurbitae* (Coquillett)	5	0	2	0	7
*Bactrocera dorsalis* (Hendel)	4	1	1	5	11
*Ceratitis capitata* (Wiedemann)	1	0	0	0	1

## Discussion

Comparative genomics is based on the similarity of the genomes of related organisms. The genomes of two or more organisms with a relatively close common ancestor have species differences between them evolved from the ancestral genome, and the closer the two organisms are in terms of evolutionary stages, the higher their genomic relatedness (Zhao, 2021). The comparative analysis of similar genomes reveals potential gene functions and elucidates species evolutionary relationships. The comparative genome discipline was used for the identification of new species. Chen et al. (2021) comparatively analyzed the chloroplast genomes of 13 species of oil teas and found that the chloroplast genome sequences of undefined oil teas in Hainan Province and Xuwen County were identical. A total of 136 genes were annotated, including 91 protein-coding genes, 37 tRNA genes, and eight rRNA genes. Comparative genomes can also be used for the selection and breeding of superior varieties. Wei (2021) conducted a comparative genomic analysis of the genomes of 11 rice genera and made progress in the differentiation of rice genera and disease resistance genes, which laid the foundation for the development of superior varieties of disease-resistant rice. In this study, the comparative genomic analysis of the genomes of three species of harmful fruit flies can provide a scientific basis for the development of effective fruit flies control strategies.

In this study, the genomes of *Z. cucurbitae* (Coquillett), *B. dorsalis* (Hendel), and *C. capitata* (Wiedemann) were comparatively analyzed, and the GO and KEGG enrichment analyses were performed for genes specific to the three fruit fly species. Among the first 20 pathways of KEGG enrichment in *Z. cucurbitae* (Coquillett), pathways related to the immune system such as the Toll and Imd signaling pathway and natural killer cell-mediated cytotoxicity appeared, and the most enriched pathway was the Toll and Imd signaling pathway, suggesting that *Z. cucurbitae* (Coquillett) may have a strong immune functions. In *B. dorsalis* (Hendel), the MAPK signaling pathway-fly and the Wnt signaling pathway, which are related to cell growth and differentiation, are more frequent, suggesting that *B. dorsalis* (Hendel) may have a faster developmental and reproductive rate. In the KEGG enrichment of genes specific to *C. capitata* (Wiedemann), the pathways associated with human diseases such as chronic myeloid leukemia and hepatocellular carcinoma were the most frequent, followed by drug metabolism-cytochrome P450 and the metabolism of xenobiotics by cytochrome P450. P450 is an important component of a multi-functional oxidase system that functions as both an oxidase and a monooxygenase, which not only catalyzes the biosynthesis and degradation of endogenous substances to maintain the normal function of the organism but also plays a detoxifying and activating role against exogenous substances (e.g., insecticides, plant secondary substances) (Feyereisen, 2015), indicating that *C. capitata* (Wiedemann) has a higher detoxification capacity.

On the basis of this study, the SP genes among the single-copy homolog genes of three fruit fly species were screened by Ka/Ks and further subjected to GO and KEGG annotation, and the KEGG enrichment results showed that the genes were enriched to the fatty acid metabolic pathway. The biosynthesis of fatty acids is a complex multi-step reaction process consisting of acetyl-coenzyme A carboxylase (ACC), fatty acid synthase (FAS), elongase of very long-chain fatty acids (ELO), desaturase (desat or fatty acid desaturase, FAD), and fatty coenzyme A reductase (fatty acyl-CoA reductase, FAR) (Zheng et al., 2017). Interference with *ACC* gene expression decreases egg production and reproduction in *Aedes aegypti* (Alabaster et al., 2011), deletion of the *FAS* gene decreases reproduction in *Nilaparvata lugens* (Stal) (Li et al., 2016), and RNA interference with the *desatF* and *eloF* genes, respectively, increases the mating duration and reduces courtship and mating attempts in male fruit flies (Chertemps et al., 2007). FAR is a key enzyme for pheromone synthesis in insects (Hagström et al., 2012), and fatty acid catabolism is one of the main energy sources of the organism; therefore, the fatty acid metabolic pathway plays an important role in the development and reproduction of insects. In the present study, SP single-copy homolog genes of three fruit fly species were used for the fatty acid metabolism pathway, indicating that this gene is a recently and rapidly evolving gene, which is closely related to the development and reproduction of fruit flies.

## Data Availability

The original contributions presented in the study are included in the article/Supplementary Material, further inquiries can be directed to the corresponding author.
